# Moya-Moya Disease Revealed by a Non-lobar Intracerebral Hemorrhage in an Adult

**DOI:** 10.7759/cureus.52204

**Published:** 2024-01-13

**Authors:** Hamid Ziani, Siham Nasri, Imane Kamaoui, Imane Skiker

**Affiliations:** 1 Radiology, Mohammed VI University Hospital, Faculty of Medicine, University Mohammed First, Oujda, MAR

**Keywords:** moyamoya, intracranial hemorrhage, ct, digital subtraction angiography, stroke

## Abstract

Moya-Moya disease (MMD) is a rare cerebral vasculopathy affecting children and adults. It is a progressive steno-occlusive arterial disease generally discovered during the etiological assessment of an ischemic or hemorrhagic stroke. Its diagnosis is based essentially on imaging. Cerebral digital subtraction angiography (DSA) remains the gold standard. We report the case of a 42-year-old male patient admitted for the loss of consciousness with a Glasgow Coma Scale (GCS) of 12/15. A brain CT scan revealed a right capsulo-lenticular hematoma with ventricular flooding and hydrocephalus. Cerebral CT angiography showed features of Moya-Moya vasculopathy, which was confirmed by a cerebral catheter angiogram.

## Introduction

Moya-Moya disease (MMD) is a rare progressive steno-occlusive intracranial vasculopathy. It was first described by Takeuchi and Shimizu in 1957. It is linked to the stenosis of the terminal portion of the internal carotid artery and its intracranial branches, with the development of abnormal collaterals, leading to ischemic and hemorrhagic complications [1]. The estimated annual incidence of Moya-Moya disease is 0.35-0.94 per 100,000 in Japan with a first peak around five years and another in the fourth decade. Affected children often develop ischemic complications, whereas adults tend to present hemorrhagic manifestations [2]. Diagnosis is based on angiography, which shows arterial stenosis and a developed collateral network with a puff of smoke appearance. The diagnostic criteria were established in 1978 and have been revised five times since then, most recently in 2021 [3].

Through this case report, we would like to show concordance between CT scan angiography and catheter cerebral angiogram in demonstrating signs of Moya-Moya disease in our patient. We would also show that deep cerebral hematomas, often of hypertensive origin, can also be seen in MMD.

## Case presentation

A 42-year-old male patient with no previous medical history presented with intense headaches of abrupt onset with vomiting. At the emergency room, the patient was unconscious with a Glasgow Coma Scale of 12/15. Blood pressure was 160/100 mmHg. An initial cerebral CT scan revealed a right capsulo-lenticular hematoma with ventricular flooding and hydrocephalus. The patient underwent external ventricular drainage the same day.

Given the patient's young age and the absence of a history of hypertension, an underlying vascular origin was suspected despite the non-lobar location of the hematoma, and a cerebral angioscan was performed one day later.

**Figure 1:**
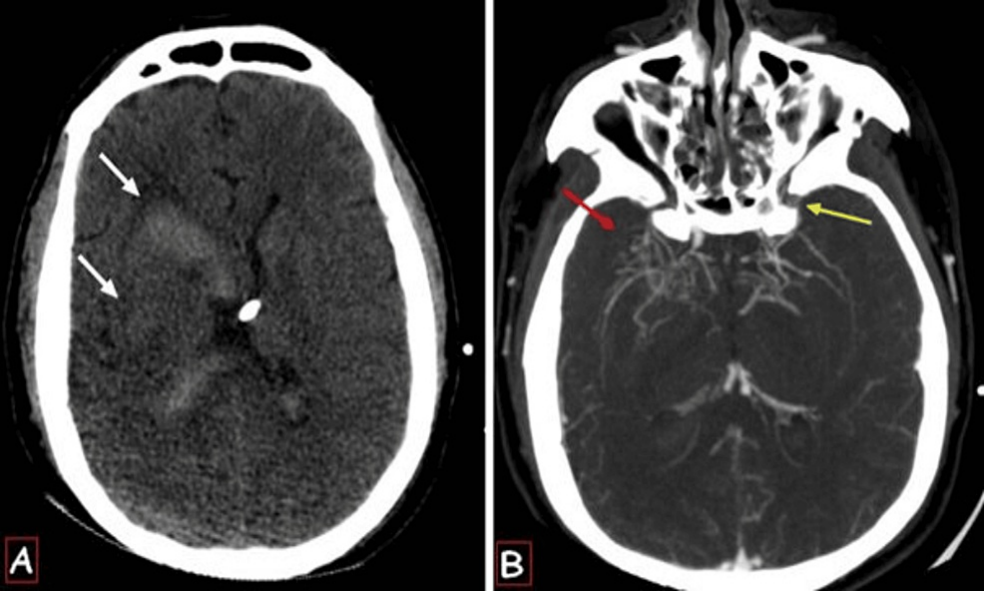
Axial brain non-contrast CT scan (A) showing a right capsulo-lenticular hematoma (white arrows) with ventricular flooding. CT angiography (B) showing the bilateral stenosis of the terminal portion of the internal carotid artery, middle and anterior cerebral arteries with a more pronounced lenticulostriate collateral network on the right side (red arrow), and abnormal collateral vessels from the branches of the external carotid artery on the left side (yellow arrow).

Cerebral angioscan showed the bilateral stenosis of the terminal portion of the internal carotid artery, middle and anterior cerebral arteries with a more pronounced lenticulostriate collateral network on the right side, and abnormal collateral vessels from the branches of the external carotid artery on the left side (Figure 1). The CT scan showed no signs of ischemia, arteriovenous malformation, or venous thrombosis, which could be potential differential diagnoses.

**Figure 2:**
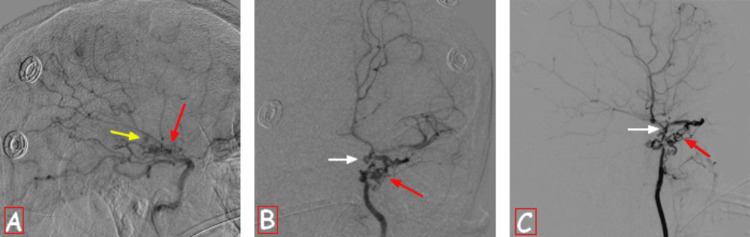
Cerebral DSA showing the stenosis of the terminal portion of the right internal carotid artery and its branches with an abnormal collateral network (red arrows) and a lenticulostriate aneurysm (yellow arrow). It also shows the stenosis of the terminal portion of the left internal carotid artery (B and C) (white arrows) with abnormal collateral vessels from the branches of the external carotid artery (red arrows). DSA: digital subtraction angiography

The patient subsequently underwent digital subtraction angiography (DSA) two days later, which confirmed the abnormalities found on the CT scan and also showed an aneurysm within the lenticulostriate network opposite the area of hematoma (Figure 2). These findings led to the diagnosis of Moya-Moya disease. There was no evidence of arteriovenous malformation or dural fistula.

During the initial phase, therapeutic management was based essentially on controlling blood pressure and intracranial pressure, as well as the prevention of seizure and thromboembolic complications. Temperature and blood glucose were also controlled. After two weeks, the patient's consciousness improved, and the follow-up CT scan showed partial resorption of the hematoma, with the regression of ventricular flooding. The decision was to perform a follow-up arteriogram after two months and prepare the patient for surgical treatment by direct bypass.

## Discussion

Moya-Moya disease (MMD) is a chronic progressive vasculopathy characterized by the stenosis of the terminal portion of the internal carotid artery and its intracranial branches, leading to the development of an abnormal collateral arterial network. This disease is more prevalent in East Asia, and its incidence has been rising over the years according to Asian and US data [1]. The estimated annual incidence of Moya-Moya disease is 0.35-0.94 per 100,000 in Japan with a first peak around five years and another in the fourth decade [2].

Clinical symptoms of MMD vary from headaches, seizures, and cognitive decline to signs of ischemic or hemorrhagic stroke. Fatigue, stress, dehydration, and infections are decompensation factors [4]. In Asian populations, affected children often develop ischemic complications, whereas adults tend to present hemorrhagic manifestations [2].

The diagnostic criteria were established in 1978 and have been revised five times since then, most recently in 2021. Minimally invasive or noninvasive angiography is the cornerstone of diagnosis [3]. The angiographic appearance varies with the stage of the disease according to the Suzuki classification. There is a correlation between the angiographic stage and clinical complications.

The non-contrast CT scan, as a first-line examination, shows ischemic or hemorrhagic complications of the disease. CT angiography detects stenotic arterial segments and the abnormal collateral network at the skull base.

MRI has also shown its role in detecting arterial stenosis and the collateral network using the three-dimensional time-of-flight (3D TOF) sequence with an accuracy of 98%-100%. Indirect MRI signs have been described, such as flow voids in the basal ganglia and/or periventricular white matter and the "ivy sign," which refers to a vivid leptomeningeal post-contrast enhancement or a high fluid-attenuated inversion recovery (FLAIR) signal intensity most likely reflecting the slow retrograde flow of the pial collaterals. Susceptibility sequences show the "brush sign," which refers to prominent deep medullary veins reflecting increased oxygen extraction in response to cerebral hypoperfusion [2,5]. Perfusion imaging (CT and MRI) shows hypoperfused cerebral territories likely to be salvageable by a revascularization procedure and allows post-reperfusion cerebral hemodynamic evaluation [6].

Digital subtraction angiography (DSA) remains the reference investigation and the gold standard for the diagnosis of MMD. According to the 2021 criteria, the diagnosis is made when the following signs are present, either unilaterally or bilaterally: stenosis or occlusion in the arteries centered on the terminal portion of the intracranial internal carotid artery and abnormal collateral network adjacent to the occlusive or stenotic lesions in the arterial phase [3].

DSA determines the stage and therefore the severity of the disease according to the Suzuki classification. It allows better study of transdural collaterals, which are indicative of advanced disease and have prognostic value in terms of perioperative ischemia and postoperative evolution [7]. Subtraction angiography detects also intracranial aneurysms whose prevalence is estimated at 14% and that are associated with a significant increase in hemorrhagic risk.

The treatment of Moya-Moya disease is mainly surgical, consisting of increasing cerebral blood flow through the creation of direct anastomoses between the external and the internal carotid system or pial synangiosis. Conservative treatment is controversial and is proposed by some teams in asymptomatic patients [8].

In the absence of any preventive treatment, the annual risk of stroke is 3.5%-5% per year, and hemorrhagic forms of MMD are of poorer prognosis compared to ischemic forms [1].

## Conclusions

The diagnosis of Moya-Moya disease is based on angiographic imaging criteria. It is revealed by intracranial ischemia or hemorrhage, which may involve different territories. Non-lobar intracranial hemorrhage can be a revealing feature of this disease. The analysis of the branches of the external carotid artery is crucial in the presence of intracerebral hemorrhage, to look for both signs of dural fistula and a possible collateral network in the context of MMD. Since the signs of MMD in the initial stages of the Suzuki classification are discrete, attention must be paid to the analysis of the carotid endings in particular.
